# Effect of clamping force on distortion of the optical surface of monochromators during assembly

**DOI:** 10.1107/S1600577522003149

**Published:** 2022-04-22

**Authors:** Eleanor Victoria Bainbridge, Jonathan David Griffiths, Jessica Clunan, Peter Docker

**Affiliations:** a School of Engineering, University of Lincoln, Brayford Way, Brayford Pool, Lincoln LN6 7TS, United Kingdom; b Diamond Light Source, Diamond House, Harwell Science and Innovation Campus, Fermi Avenue, Didcot OX11 0DE, United Kingdom

**Keywords:** silicon monochromators, finite-element analysis, clamping distortion

## Abstract

As Diamond Light Source embraces the move towards becoming a fourth-generation light source its optics will be required to perform under increasingly demanding conditions. Foremost amongst these conditions will be the increasing power densities the optics are subjected to and the reducing real estate they have to perform in. With these new challenges comes the need for greater understanding of how optics are assembled and how consistently the activity is carried out. In this paper, the effect of bolt pretension during assembly of monochromators on distortion of the optical surface is investigated through numerical simulation. The results reveal skewed convex distortion of the optical surface in the meridional direction when uneven clamping force is applied, highlighting the importance of taking the potential for distortion of the optical surface due to clamping force into consideration.

## Abstract 2

This work demonstrates the effects of clamping force on the optical surface of a monochromator silicon crystal through the use of finite-element analysis.

## 1. Introduction

Synchrotron radiation is used for the investigation of the structure and properties of a wide range of materials, with applications including analysis of engineering components, pharmaceutical design and archaeology (Yan *et al.*, 2021 ▸; Nistea *et al.*, 2019 ▸). To enable new science and research progress in all areas of operation, Diamond is moving toward the Diamond-II upgrade, a co-ordinated programme of development that combines a major machine upgrade with new instruments and complementary improvements to optics, which will be required to perform to greater accuracy with reduced tolerances (Yan *et al.*, 2021 ▸).

Single-crystal silicon monochromators are important diffractive optical components in a synchrotron beamline, used to select a particular wavelength. One common configuration for a monochromator assembly comprises a silicon crystal mounted in a cryogenic heat exchanger with through bolts threaded through the centre of the crystal to (i) hold the assembly together, and (ii) provide sufficient contact pressure between the crystal and heat exchanger to facilitate effective heat transfer. Deformation of the optical surface of the crystal will result in a change in the orientation of the crystal lattice, resulting in a change in wavelength in the reflected beam (González, 2012 ▸). Distortion due to incorrect assembly or thermal warping can result in higher beam divergence and a reduced capacity to focus the beam (Edwards *et al.*, 1985 ▸). To meet the required tolerance in terms of monochromaticity and collimation of the diffracted beam, optical surfaces with sub-nanometre figure errors are required (Alcock *et al.*, 2010 ▸; Sawhney *et al.*, 2010 ▸).

In recent years, extensive research has been carried out on the distortion of a crystal monochromator caused by thermal loading from synchrotron radiation. Chumakov *et al.* (2014 ▸) investigated the performance of a silicon monochromator under high heat load. The optical surface slope error was found to have the largest magnitude of influence on the reflected radiation as opposed to the deformation of the bulk volume. Brumund *et al.* (2021 ▸) analysed the performance of a liquid-nitro­gen-cooled high-heat-load monochromator through use of coupled thermal–mechanical finite-element simulations. The optimum heat load to minimize the thermal sagittal and meridional slope errors on the optical surface was determined. Comparatively little is understood regarding the effect of the assembly process on the divergence and spatial extent of resulting beams in X-ray optics (Hart, 1996 ▸). Siewert *et al.* (2012 ▸) demonstrated experimentally how clamping forces can result in distortion of optical surfaces. The potential for distortion of the optical surface induced by a top-end clamping arrangement was investigated experimentally by Cocco *et al.* (2001 ▸). Distortion of the optical surfaces of silicon X-ray optics caused by mounting in cryogenic heat exchanger assemblies was considered in thermal–mechanical simulations conducted by Volpe *et al.* (2018 ▸). The fastening process was shown to increase the slope error. Nistea *et al.* (2019 ▸) observed the effects of mechanical clamping on the overall performance of plane grating monochromators on the I21 beamline at Diamond. The optical surfaces of the monochromator were profiled and found to have a slope error of approximately 0.25 µrad. Through an iterative process of re-clamping, slope errors of approximately 0.1 µrad to 0.15 µrad were achieved. Stimson *et al.* (2017 ▸, 2019 ▸) investigated the potential to utilize an interference fit between a cylindrical crystal and copper heat exchanger at cryogenic temperatures to produce repeatable contact pressures at the thermal interface.

In this paper, the effect of side clamping with the use of through bolts and clamping force applied during the assembly of monochromators on the distortion of the optical surface is investigated through numerical simulation, including the effect of uneven clamping force, through analysis of surface profiles and sagittal and meridional slope errors. The potential for angular distortion in the reflected beam as a result of this distortion is discussed.

## 2. Finite-element model development

A static structural finite-element model of a monochromator assembly used on the I20 beamline at Diamond was created using ANSYS 19.2, as detailed in the following subsections.

### 2.1. Geometry

**Figure 1:**
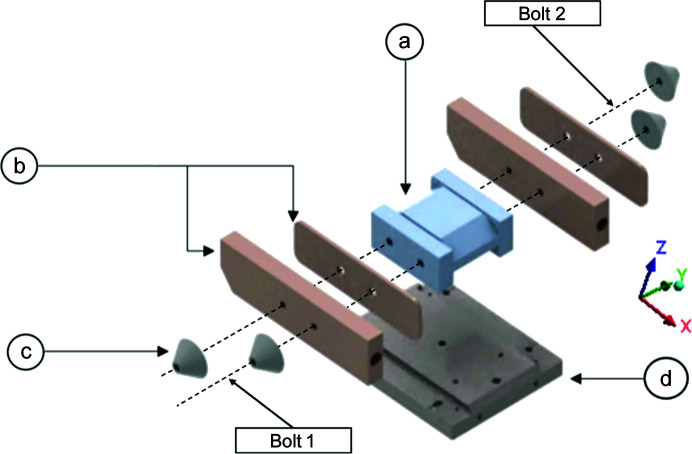
Exploded view of the monochromator assembly with (*a*) a silicon crystal, (*b*) copper cooling blocks with side panels, (*c*) cones to ensure even distribution of clamping force and (*d*) a base plate. The assembly is clamped with bolts using the through holes. The bolt locations are represented by dashed lines.

The monochromator assembly consists of a silicon crystal flanked by two copper heat exchangers on either side. Liquid-nitro­gen coolant flows through channels in these heat exchangers to facilitate effective heat transfer from the optical surface of the crystal to the surroundings. Thin high-purity indium foil is sandwiched between the crystal and copper heat exchangers to improve contact and thermal contact conductivity, modelled using a surface body with the thickness set to 0.5 mm. The assembly is held together by two steel through bolts, threaded through the centre of the crystal. On either side of the assembly there are cone-shaped structures which ensure a more evenly distributed force across the contact faces. An exploded view of the monochromator assembly is shown in Fig. 1 ▸.

Material properties for steel, anisotropic silicon, copper alloy and indium foil were taken from built-in libraries in ANSYS and the NIST database (https://trc.nist.gov/cryogenics/materials/materialproperties.htm). The anisotropic stiffness coefficients for the Si(100) crystal plane are used, with the effect of crystal orientation given no further consideration (Zhang *et al.*, 2014 ▸).

### 2.2. Boundary conditions

Boundary conditions were applied to effectively simulate the assembly process. This includes a fixed support on the bottom surface of the base plate, with bolt pretension applied using two line bodies to represent the through bolts. The ends of each line body are scoped to the appropriate face of the cones on either side of the assembly shown in Fig. 1 ▸ using joints. Cylindrical supports are applied to the inner cylindrical faces of the through holes. An acceleration load is applied to simulate the effect of gravity, acting in the negative *z* direction. The beam propagation direction is in the *xz* plane in Fig. 1 ▸. Frictionless contact formulation is specified at all silicon, copper and indium interfaces.

### 2.3. Meshing

A meshing strategy was adopted which ensured high-quality elements, numerical accuracy and high computational efficiency. A hex-dominant method was applied to the two copper blocks and to the crystal, with a body sizing of 7.5 mm. A face sizing of 0.99 mm was applied to the optical surface of the crystal as this is the principal region of interest. A face-mapping was specified on the faces of the cones to ensure sufficient element quality.

## 3. Results and discussion

The numerical modelling work focus was the effect of bolt pretension – applied to the two through bolts in the assembly – on the distortion of the optical surface of the crystal. During assembly, the bolt pretension must satisfy the following conditions: (i) it should be large enough to ensure good contact (and uniformity of contact) between the crystal and heat exchanger walls to facilitate effective heat transfer from the crystal to the atmosphere during service (thereby minimizing thermal distortion of the optical surface); (ii) the clamping force should not be so high as to mechanically distort the optical surface.

A study was conducted in which the bolt pretension in each bolt was varied between 152 N and 228 N, ±20% of the standard applied bolt pretension of 190 N for this monochromator assembly, as specified by Diamond Light Source.

### 3.1. Effect of clamping force on distortion of the optical surface

**Figure 2:**
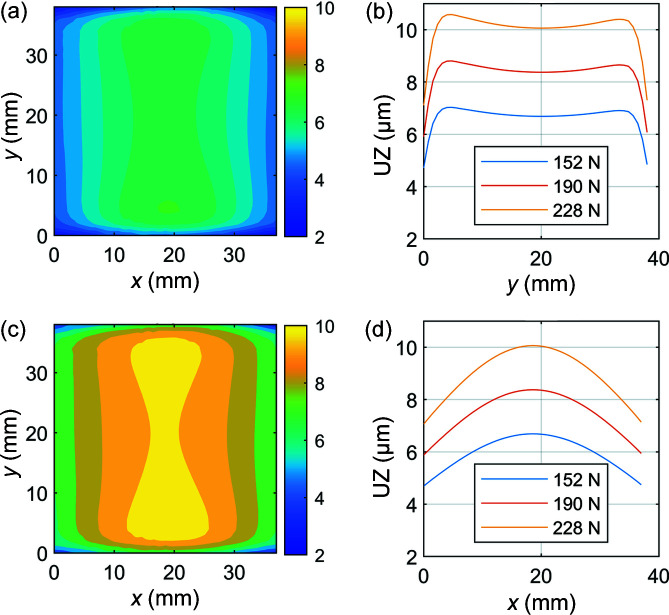
Distortion of the optical surface with (*a*) and (*c*) showing contour plots of maximum total deformation (nm) on the optical surface of the crystal for evenly applied bolt pretensions of 152 N and 228 N, respectively; and (*b*) and (*d*) showing vertical displacement UZ across the centre line of the optical surface of the crystal in the sagittal and meridional directions, respectively.

To determine the effect of clamping force on the distortion of the optical surface, three simulations were conducted in which an equal bolt pretension was applied to the two bolts in the assembly: 152 N, 190 N and 228 N. The resulting distortion of the optical surface is shown in Fig. 2 ▸.

Fig. 2 ▸ reveals an increase in the magnitude of distortion of the optical surface with increasing clamping force. This is reflected in the meridional and sagittal slope errors, shown in Fig. 4. Slope errors were obtained by taking height profile derivatives in the meridional and sagittal directions across the centre line of the optical surface.

**Figure 3:**
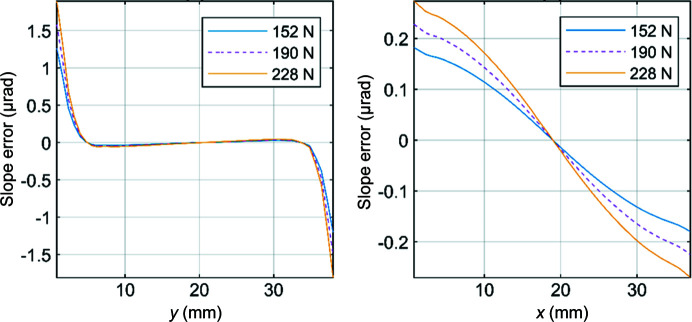
Slope errors across the centre line of the optical surface of the crystal in the (*a*) sagittal direction and (*b*) meridional direction.

**Figure 4:**
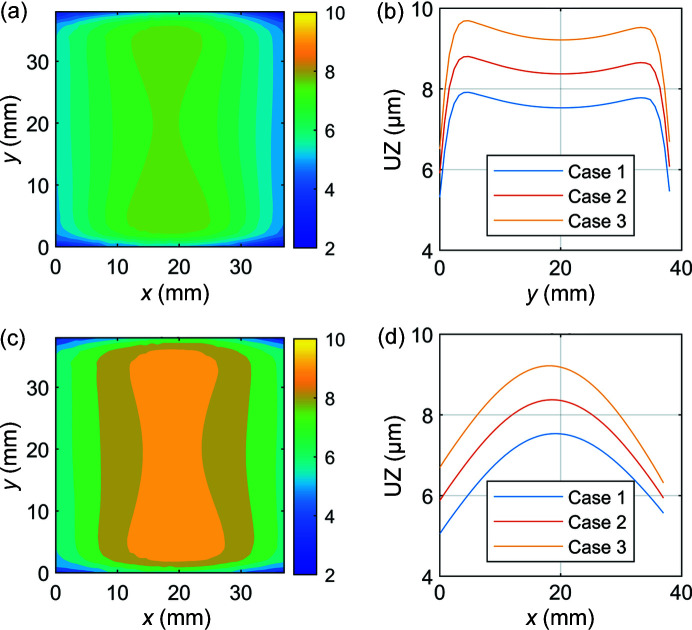
Distortion of the optical surface with (*a*) and (*c*) showing contour plots of maximum total deformation (nm) on the optical surface of the crystal for Case 1 and Case 3, respectively; and (*b*) and (*d*) showing vertical displacement UZ across the centre line of the optical surface of the crystal in the sagittal and meridional directions, respectively.

Figs. 3 ▸(*a*) and 3(*b*) show the sagittal and meridional slope errors increasing with clamping force, respectively. This effect is more prominent in the case of meridional slope error in Fig. 4 ▸(*b*). This can be attributed to the nature of the distortion profiles shown in Fig. 2 ▸, in which variation is greater in this direction. This is significant, as meridional slope errors have a greater influence on angular distortion of the beam compared with sagittal slope errors (Brumund *et al.*, 2021 ▸). The peak-to-valley of the figure error and RMS slope errors across the entire centre line of the optical surface in the meridional and sagittal directions are summarized in Table 1 ▸. These values are consistent with those reported by Nistea *et al.* (2019 ▸).

### 3.2. Effect of uneven clamping force

The effect of different applied bolt pretensions between bolts 1 and 2 shown in Fig. 1 ▸ was determined by considering three load cases:

1. Case 1: bolt 1 tightness reduced by 20% to 152 N, bolt 2 remains at 190 N.
2. Case 2: equal 190 N bolt pretention applied to bolts 1 and 2.
3. Case 3: bolt 1 tightness increased by 20% to 228 N, bolt 2 remains at 190 N.

The distortion of the optical surface for each of these cases is shown in Fig. 4 ▸.

**Figure 5:**
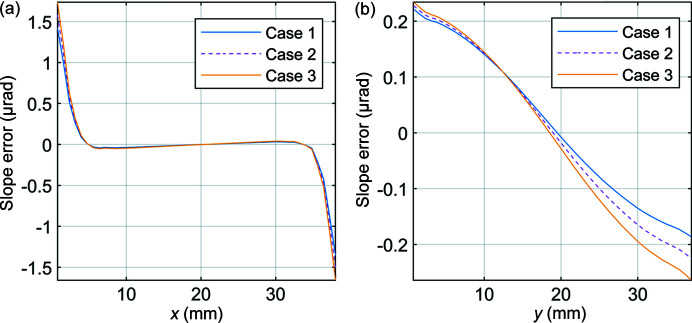
Slope errors across the centre line of the optical surface of the crystal in the (*a*) sagittal and (*b*) meridional directions.

Considering the meridional direction due to its greater influence on angular distortion of the beam, it is clear from Fig. 4 ▸ that the effect of uneven bolt pretension is to produce a skewed convex distortion profile on the optical surface, with the magnitude of distortion increasing with total clamping force. The skewed nature of the distorted surface profile can be attributed to torsion of the optical surface. The sagittal and meridional slope errors for each case are shown in Fig. 5 ▸.

Figs. 5 ▸(*a*) and 5(*b*) reveal a reduction in sagittal and meridional slope error for Case 1 (under-tightened) and an increase for Case 3 (over-tightened), relative to Case 2 (even bolt pretension). This is consistent with the observation in Section 3.1 that total bolt pretension has a positive correlation with surface distortion and therefore slope error. The effect of different applied bolt pretensions between the two bolts is most evident in the case of meridional slope error shown in Fig. 5 ▸(*b*). With uneven bolt pretension, the maxima in the distortion on the optical surface shifts from the centre of the optical surface (and beam footprint). The direction of this shift is dictated by the difference in applied bolt pretension (that is, under-tightened or over-tightened) and will result in asymmetrical angular distortions of the subsequent beam (Brumund *et al.*, 2021 ▸). The peak-to-valley of the figure error and RMS slope errors across the entire centre line of the optical surface in the meridional and sagittal directions are summarized in Table 2 ▸.

## 4. Conclusions

In this paper, numerical simulations have been utilized to determine the effect of the assembly process – specifically clamping force applied with through bolts – on the distortion of the optical surface of the crystal in a monochromator assembly used on the I20 beamline at Diamond Light Source. We found that evenly distributed clamping force produced approximately symmetrical convex distortion of the optical surface in the meridional direction, the magnitude of which was proportional to the clamping force itself. Unevenly distributed clamping force was found to result in skewed convex distortion of the optical surface, with the maxima shifted from the centre of the optical surface (and beam footprint). This results in asymmetrical meridional slope errors of the optical surface which induce asymmetrical angular distortions of the subsequent beam. The simulation results should be validated experimentally (*e.g.* using Fizeau interferometry). Further modelling work is required to determine the effect of the assembly process on thermal management and angular distortion of the reflected beam under beamline operating conditions.

## Figures and Tables

**Table 1:** Peak-to-valley of the figure error and RMS slope errors across the centre line of the optical surface of the crystal

	RMS slope error (µrad)		Peak to valley (nm)	
Applied bolt pretension (N)	Meridional	Sagittal	Meridional	Sagittal
152	0.32	0.12	2.32	2.00
190	0.40	0.15	2.90	2.50
228	0.48	0.18	3.47	3.00

**Table 2:** Peak-to-valley of the figure error and RMS slope errors across the centre line of the optical surface of the crystal

	RMS slope error (µrad)		Peak to valley (nm)	
	Meridional	Sagittal	Meridional	Sagittal
Case 1	0.36	0.13	2.61	2.48
Case 2	0.40	0.15	2.90	2.50
Case 3	0.44	0.16	3.19	2.90
